# Case Report: The Use of Intravenous SMOFlipid Infusion to Treat Severe Asparaginase-Induced Hypertriglyceridemia in Two Pediatric Acute Lymphoblastic Leukemia Patients

**DOI:** 10.3389/fped.2021.660627

**Published:** 2021-04-22

**Authors:** Sie Chong Doris Lau, C-Khai Loh, Hamidah Alias

**Affiliations:** Pediatric Hematology and Oncology Unit, Department of Pediatrics, UKM Medical Centre, Faculty of Medicine, The National University of Malaysia, Kuala Lumpur, Malaysia

**Keywords:** acute lymphoblastic leukemia, asparaginase, hypertriglyceridemia, omega-3, SMOFlipid, pediatric, case report

## Abstract

Asparaginase-induced hypertriglyceridemia can have a spectrum of clinical presentations, from being asymptomatic to having life-threatening thrombosis or hyperviscosity syndrome. At present, there is no recommendation on routine lipid monitoring during asparaginase-containing treatment phase, nor a standardized guideline on its management. Two cases are presented here to illustrate the effects of concurrent infection on asparaginase-induced hypertriglyceridemia in patients with high-risk ALL and the use of SMOFlipid infusion as a treatment option in an acute situation.

## Introduction

Asparaginase is an important chemotherapeutic agent used in remission induction protocols for childhood acute lymphoblastic leukemia (ALL) (1, 2). However, its use has been associated with abnormalities in lipid metabolism, such as hypertriglycerides (3–5). Although, this is usually transient, it may also cause transaminitis, pancreatitis, life-threatening thrombosis, or hyperviscosity syndrome (6–11). Currently there is no standard guideline for the management of asparaginase-induced hypertriglyceridemia. Here, we report the beneficial use of SMOFlipid infusion (containing 3% fish oil which is high in eicosapentaenoic acid [EPA] and docosahexaenoic acid [DHA]) to bring down the triglyceride (TG) levels in two teenagers with severe hypertriglyceridemia following induction chemotherapy for high-risk ALL.

## Case 1

**Figure 1:**
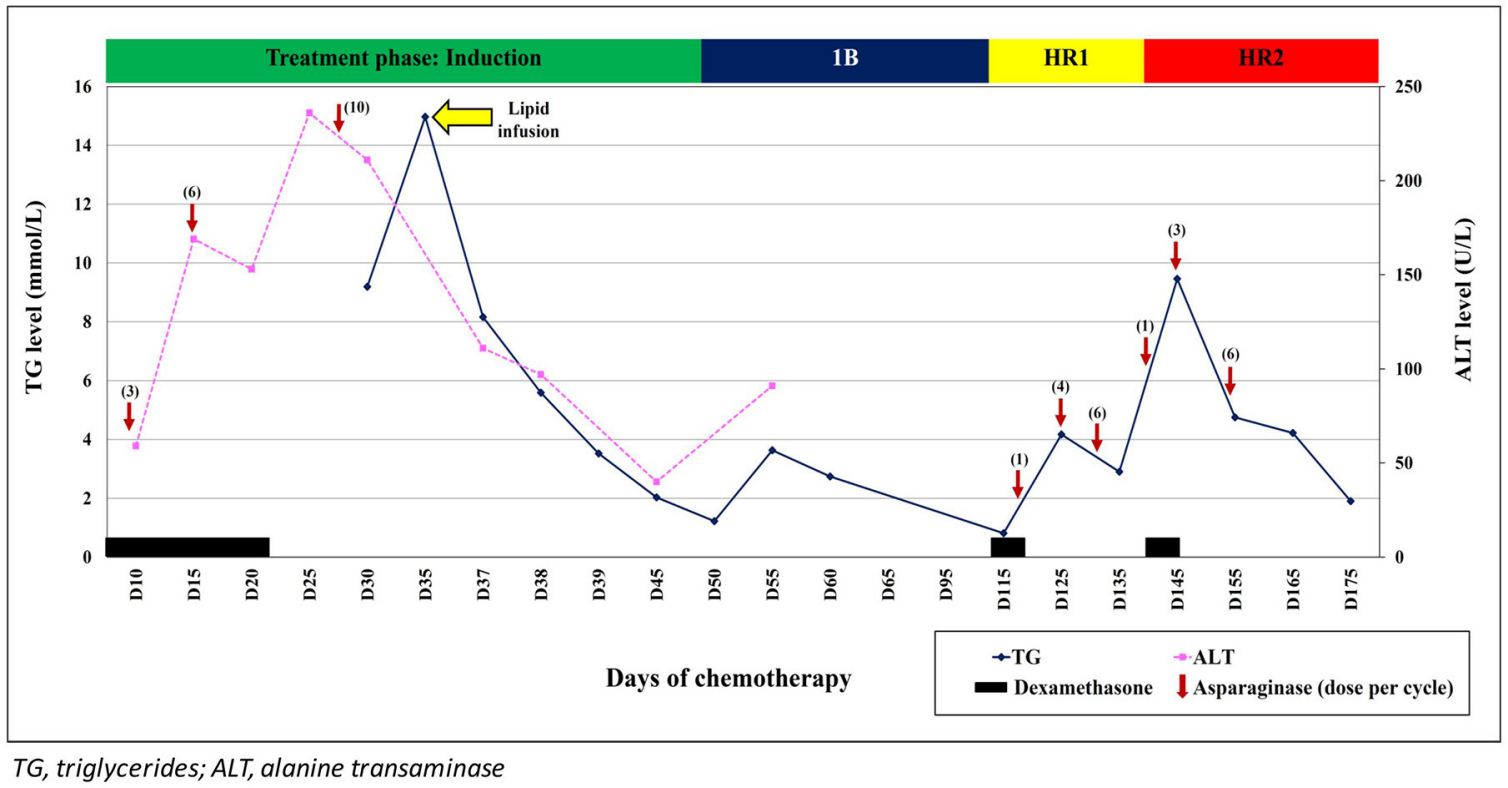
Triglyceride and alanine transaminase (ALT) trends during treatment with L-asparaginase and following infusion of SMOFlipid.

A 12-year-old boy with no family history of dyslipidaemia presented with 1-week history of fever and bilateral lower limb petechiae. Clinically, he was pale and had multiple cervical lymphadenopathy and hepatomegaly. Bone marrow aspiration and trephine biopsy (BMAT) confirmed the diagnosis of pre-B ALL and he was stratified as high risk ALL due to hyperleukocytosis (white cell count of 65.1 × 10^9^/L) at initial presentation. He was started on induction chemotherapy (UKALL 2003 Regimen B) consisting of intrathecal methotrexate (MTX), oral dexamethasone (6 mg/m^2^/day for 28 days), intramuscular (IM) *E. coli* L-asparaginase (6000 IU/m^2^ every other day for 12 doses), intravenous (IV) vincristine (1.5 mg/m^2^/dose weekly for 5 weeks) and IV daunorubicin (25 mg/m^2^/dose weekly × 4 weeks). At Day 15 induction, he developed recurrent epigastric pain; serum amylase, and ultrasound abdomen revealed no evidence of pancreatitis. His serial liver function test however showed mild elevation of alanine transaminase (ALT) ranging between 59 and 169 U/L (N:0–55 U/L). He was managed symptomatically as dexamethasone-induced gastritis with close monitoring of his liver function. Subsequently he was admitted at Day 20 induction for febrile neutropenia secondary to periungual abscess and was started on broad spectrum antibiotics. At this juncture, he also had grade IV oral mucositis and was unable to tolerate orally. Prior to commencement of total parenteral nutrition (TPN), blood investigations were performed and revealed elevated serum triglyceride (TG) at 8.86 mmol/L as well as elevated liver transaminases - ALT of 219 U/L; aspartate transaminase (AST) of 103 U/L (N: <45 U/L); gamma-glutamyl transferase (GGT) of 370 U/L (N: 13–64 U/L). Apart from the intermittent epigastric pain, he had no other complaint hence the hypertriglyceridemia was managed conservatively with low fat diet; lipid was omitted from the TPN. By this time, he had already received 10 doses of native *E. coli* L-asparaginase (Figure 1).

While in the ward, he became delirious and had incoherent speech. Clinical examination did not reveal any focal neurological deficit and his blood pressure was normal. Magnetic resonance imaging (MRI) of the brain showed no evidence of posterior reversible encephalopathy syndrome (PRES), MTX neurotoxicity, or leptomeningeal enhancement. Magnetic resonance angiography (MRA) was also performed and revealed normal cerebral vessels with no thrombosis. The cerebrospinal fluid (CSF) sent for cytology, culture, microscopy examination, and biochemistry results were all normal. However, at this point of time, his serum TG had increased further to 14.97 mmol/L. Due to unavailability of plasmapheresis service at that critical time, a decision was made to give him IV infusion of SMOFlipid at 0.5 g/kg/day. His conscious level improved within 24 h and the serum TG level reduced rapidly over the next 2 days. Lipid infusion was ceased after 5 days. His liver function normalized after 2 weeks. Subsequent chemotherapy was changed to EsPhALL protocol (Version 5) as his BCR-ABL was positive. Although, each cycle of L-asparaginase administration was associated with transient increase in the serum TG, he remained asymptomatic. Unfortunately, 6 months after diagnosis, he developed *Plesiomonas shigelloides* and *Klebsiella pneumoniae* septicaemic shock and succumbed.

## Case 2

A 13-year-old boy with late relapse B-ALL (isolated marrow relapse with BCR-ABL positive at relapse) was commenced on chemotherapy – EsPhALL protocol (Version 5)(induction phase as per UKALL 2003 Regimen B protocol, and concurrent oral Imatinib 300 mg/m^2^ daily). He had no history of severe adverse reaction toward chemotherapy during his initial treatment for standard risk pre-B ALL at 4 years old (UKALL 97(99) Regimen A). There was no family history of dyslipidaemia. The day 15 induction bone marrow and cytogenetic examination were in remission. At Day 28 induction chemotherapy, he developed severe vincristine-induced peripheral neuropathy and was admitted for supportive care. Blood investigation on admission revealed elevated liver transaminase (ALT 402 U/L); serum TG was not performed. He had received a total of 12 doses of native *E. coli* L-asparaginase as per treatment protocol. Retrospectively, his serial ALT and AST during the induction phase chemotherapy was elevated, ranging between 57–345 U/L and 91–92 U/L, respectively, while his serum TG was 1.77–3.07 mmol/L.

On day 3 of admission, he developed febrile neutropenia and *Pseudomonas aeruginosa* septicaemic shock. Broad-spectrum antibiotic was commenced and he was transferred to Pediatric Intensive Care Unit (PICU) for inotrope support. His condition stabilized and the inotrope was weaned off after 72 h. Blood culture taken after 48 h of antibiotic initiation was negative. However, while in PICU, he became delirious and started talking inappropriately. His pupils were equal and reactive bilaterally and neurological examination did not reveal any focal abnormality. Brain imaging and lumbar puncture were not performed as he was not stable at that time. The serial liver function test (LFT) showed worsening transaminitis (ALT 592 U/L) at this juncture, and fasting TG was markedly elevated (15.49 mmol/L). SMOFlipid infusion was initiated at 0.5 g/kg/day and the serum TG declined to 5.77 mmol/L in 2 days. Unfortunately, he developed *Candida tropicalis* septicaemia with multiorgan failure 3 weeks later and succumbed. Post-mortem lumbar puncture showed no evidence of meningitis.

## Discussion

Asparaginase-induced hypertriglyceridemia has been reported to occur in 10–67% of children treated for ALL (4, 6, 8). As routine lipid monitoring is not incorporated into most of the treatment protocol worldwide, this condition could be underdiagnosed. In patients with unexplained transaminitis, a high index of suspicion would allow prompt investigations for hypertriglyceridemia as illustrated in the cases above. Both our patients most likely had asparaginase-induced hypertriglyceridemia during the early phase of induction chemotherapy but were clinically asymptomatic. We postulate that the acute febrile illness exacerbate the existing problem of hypertriglyceridemia, leading to rapid increase in the TG levels and this could cause hyperviscosity. Comprehensive investigations are required for any patient who presents with neurological impairment in order to exclude other differential diagnoses including meningitis, encephalitis, and intracranial bleed. In oncology patients, MRI brain, whenever possible, should be performed to look for changes suggestive of MTX neurotoxicity, or PRES which may also present with altered mental status before attributing it to hypertriglyceridemia.

As hypertriglyceridemia is transient and majority of the patients are asymptomatic, no omission, or modification of the asparaginase dosage are required (5, 6, 8). However, symptomatic patients or those with markedly elevated TG level may benefit from medical treatment. There are currently no standard treatment guidelines for asparaginase-induced hypertriglyceridemia in pediatric patients with ALL. Isolated case reports and small reviews have suggested several approaches, namely short-term fasting or low-fat diet, oral fibrates, oral omega-3 fatty acids, and insulin infusion (7, 10, 12, 13). Plasmapheresis has been shown to be effective in reducing TG levels rapidly after 2 h of apheresis (9); clinical improvement was seen as early as 8 h and normalization of the TG levels was achieved after 72 h of plasmapheresis (3). However, the availability of plasmapheresis may be limited due to the cost and requirement for technical expertise. The use of SMOFlipid infusion to treat severe or symptomatic hypertriglyceridemia has not been reported before.

SMOFlipid is a lipid emulsion formulation which has been used as a source of parenteral nutrition for pediatric patients with critical illness, following major gastrointestinal tract surgery or preterm babies who were unable to tolerate enteral feeding (14, 15). Small number of studies has shown that it was safe and well-tolerated among the pediatric population (16–18). A further study on the safety and efficacy of SMOFlipid in pediatric patients aged 3 months to 16 years old is currently ongoing (www.clinicaltrials.gov Identifier: NCT03563222). The constituents of SMOFlipid are soybean oil (6%), medium chain triglycerides (6%), olive oil (5%), and fish oil (3%). The fish oil here is characterized by a high content of eicosapentaenoic acid (EPA) and docosahexaenoic acid (DHA) which are long-chain, polyunsaturated, omega-3 fatty acids. Although the exact mechanisms of action are not well-understood, it was proposed that omega-3 fatty acids can reduce hepatic very-low-density lipoprotein (VLDL)-TG synthesis/secretion and enhances TG clearance from circulating VLDL and chylomicron particles (19, 20). The use of oral omega-3 fatty acids in addition to dietary modification in a relatively well patient allows reduction of TG levels over a slower course (6, 12). As both our patients were unwell and had severe hypertriglyceridemia, an alternative approach was considered in the absence of plasmapheresis. The idea of using SMOFlipid came from the afore-mentioned principle that the omega-3 (fish oil) in the SMOFlipid can also theoretically be used to reduce triglyceride level. Precaution must be taken as SMOFlipid is contraindicated in severe hyperlipidaemia or severe disorders of lipid metabolism with serum TG >1,000 mg/dL. However, our patients did not have these comorbidities prior to the illness hence, SMOFlipid infusion was initiated at the lowest infusion rate of 0.5g/kg/day with close monitoring to ensure no further increment of the TG level. Clinical improvement was seen within 24–48 h concurrent with the reduction of TG level although biochemical markers abnormalities (liver transaminases) may persist for several weeks. Subsequent asparaginase doses may be given safely with close monitoring of the TG levels as in our first patient (21).

The two cases here highlighted the importance of early recognition of clinical and biochemical indicators of hypertriglyceridemia in patients with high-risk ALL during induction chemotherapy, especially in the setting of acute infection. We also reported our observation on the effect of SMOFlipid infusion in lowering the TG level rapidly in acute situation when plasmapheresis is not available at the treating centre. However, further study regarding feasibility and safety of SMOFlipid would be required before it can be recommended as one of the treatment option for hypertriglyceridemia.

## Data Availability Statement

The raw data supporting the conclusions of this article will be made available by the authors, without undue reservation.

## Ethics Statement

Written informed consent was obtained from the individual(s), and minor(s)' legal guardian/next of kin, for the publication of any potentially identifiable images or data included in this article.

## Author Contributions

SL, C-KL, and HA were responsible for the clinical management of the patients. SL acquired the clinical data and drafted the initial manuscript. HA reviewed the intellectual contents of the manuscript and made substantial modification during revision. All authors reviewed and approved final manuscript as submitted and agree to be accountable for all aspects of the work.

## Conflict of Interest

The authors declare that the research was conducted in the absence of any commercial or financial relationships that could be construed as a potential conflict of interest.
